# Book Notices

**Published:** 1868-06

**Authors:** 


### Book Notices.

Therapeutics and Materia Medica. A Systematic Treatise on the Action and Uses of Medicinal Agents, Including their Description and History. By ALFRED STILLE, M.D., Prof. Theory and Practice of Medicine, and of Clinical Medicine, in the University of Pennsylvania; Physician to St. Joseph's Hospital and to the Philadelphia Hospital; etc., etc., etc. Third Edition, Revised and Enlarged. In Two Volumes. Philadelphia: HENRY C. LEA. 1868.

This, third edition of Dr. Stillé's very complete and valuable work on Therapeutics and Materia Medica, comes to us in two volumes, of over 800 pages each, good type, and good leather binding. The author seems to have carefully revised the whole work; and has treated of the following substances, not found in the previous editions, *viz.:* chromic acid, permanganate pot-assa, sulphites of soda, etc., carbolic acid, nitrous oxide, rhigolene, and calabar bean. It is certainly one of the best works on Materia Medica and Therapeutics in our language. For sale by W. B. KEEN & Co., 148 Lake Street, Chicago.

Odontalgia, commonly called Toothache: Its Causes, Prevention, and Cure. By S. PARSONS SHAW. Philadelphia: LIPPINCOTT & Co. 1868.

This is a neatly published duodecimo volume, of 258 pages. Its title clearly indicates the subjects treated in the work. It is written in pleasing style, and will well repay both physician and dentist for a careful perusal. For sale by S. C. GRIGGS & Co., Chicago.

Archives de Physiologie Normale et Pathologique. Publiérs par MM. BROWN-SEQUARD, CHERCOT, VULPIAN. No. 2 Mers. April, 1868. Avec 5 Planches. Paris: VICTOR MASSON et FILS, Place de L'Ecole de Médicine.

This is the title to the second number of a bi-monthly journal, devoted to physiology and pathology, published in Paris, by Brown-Séquard, Chercot, and Vulpian. Each number contains about 200 pages, and is illustrated by plates of the highest finish. To all who read the French language with facility, this would be an exceedingly interesting and valuable periodical.

